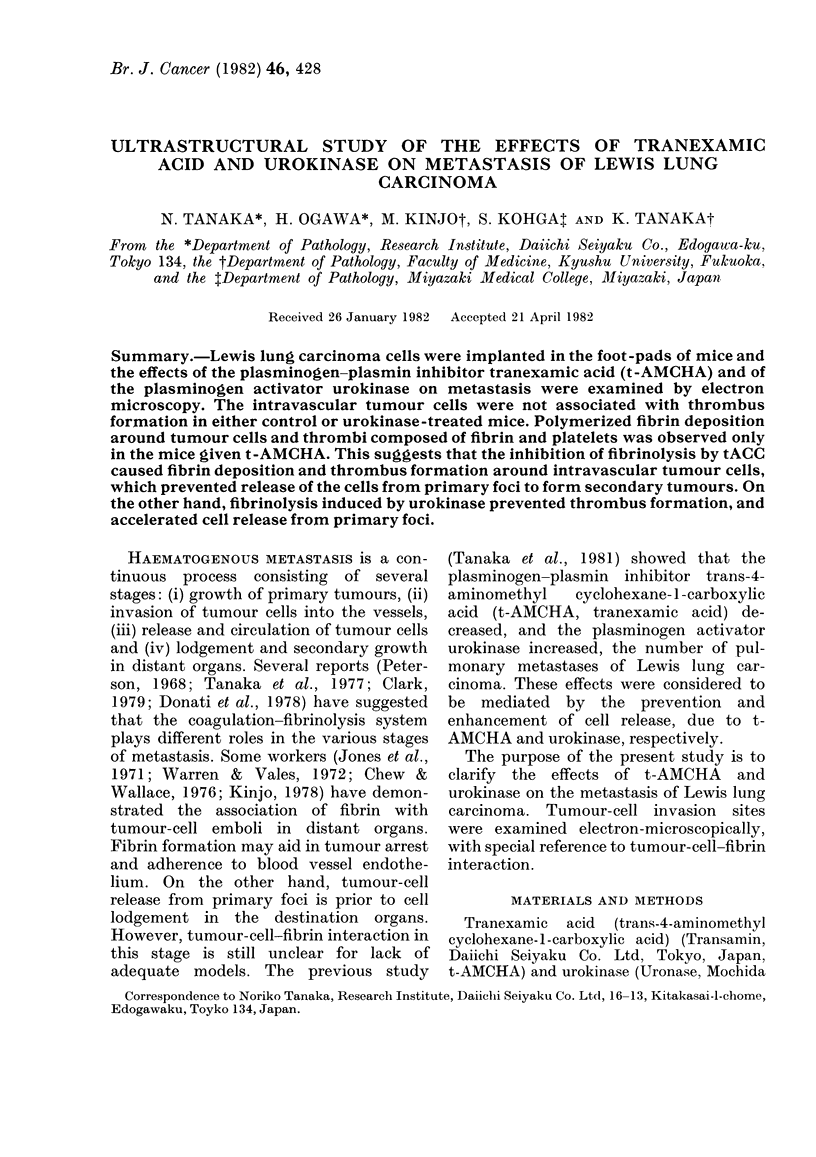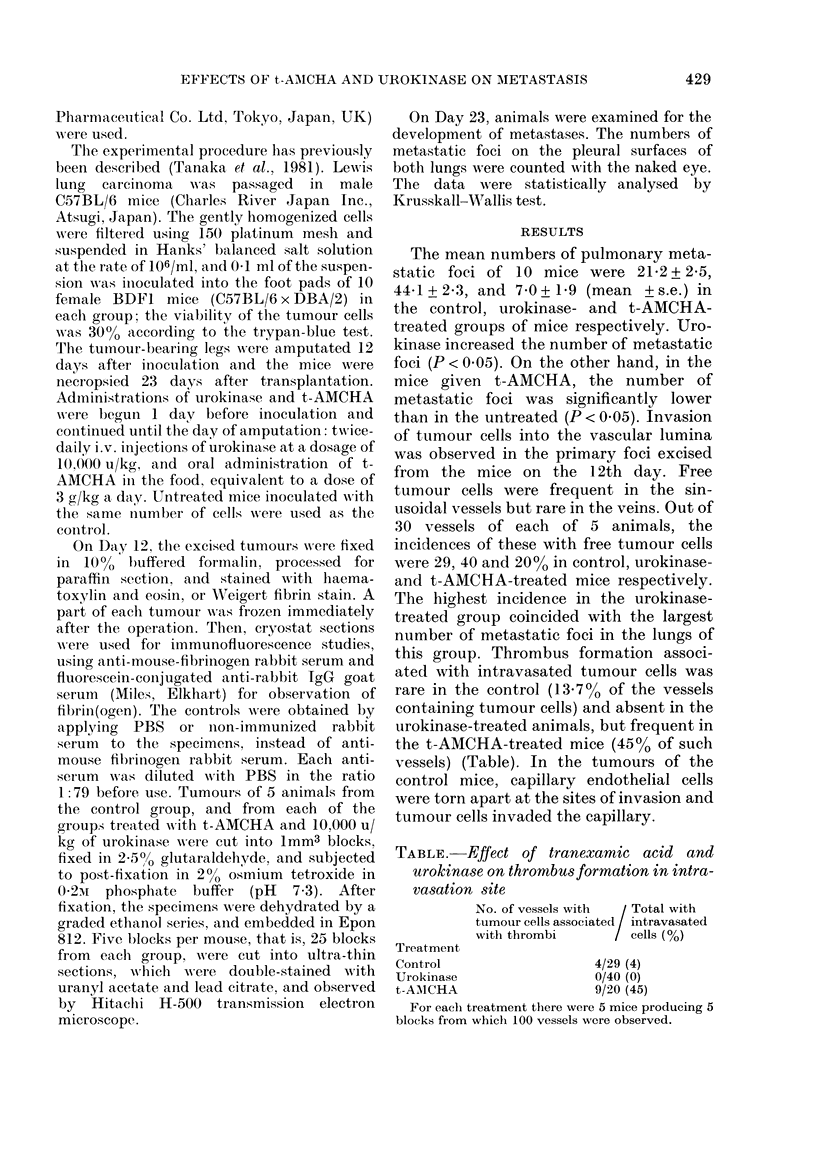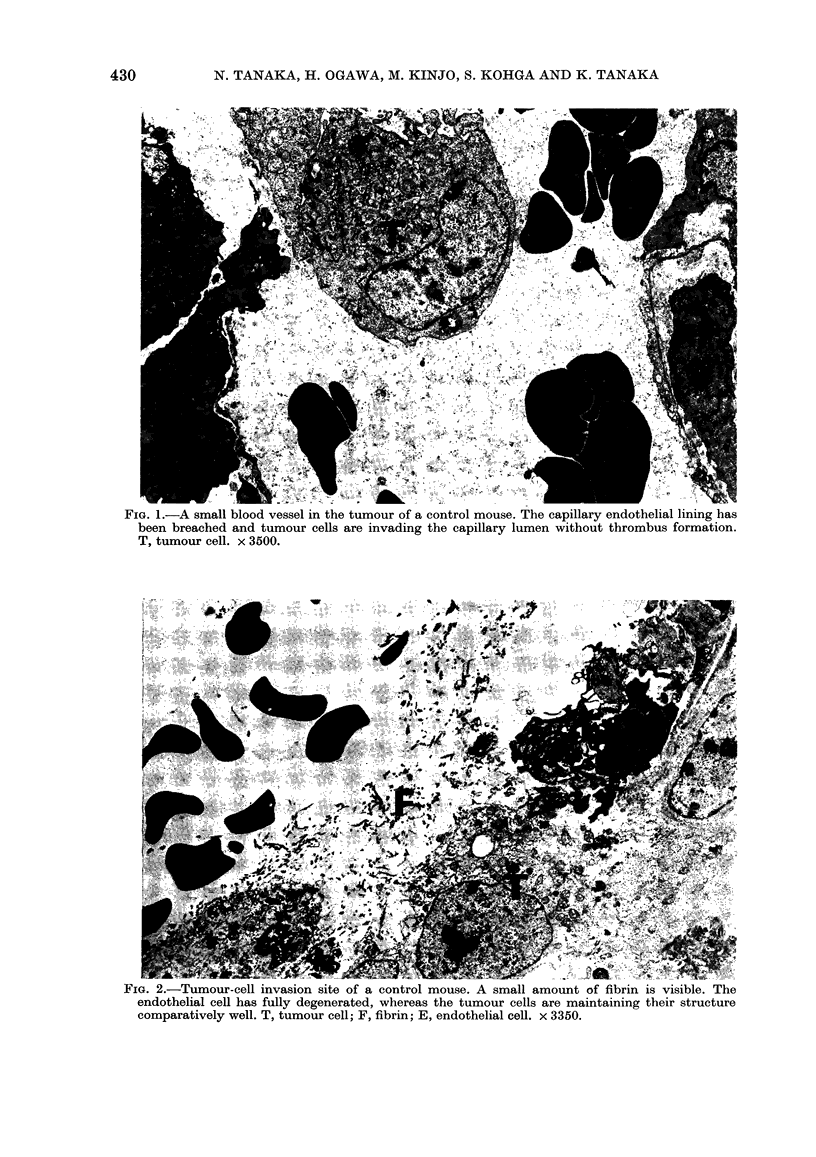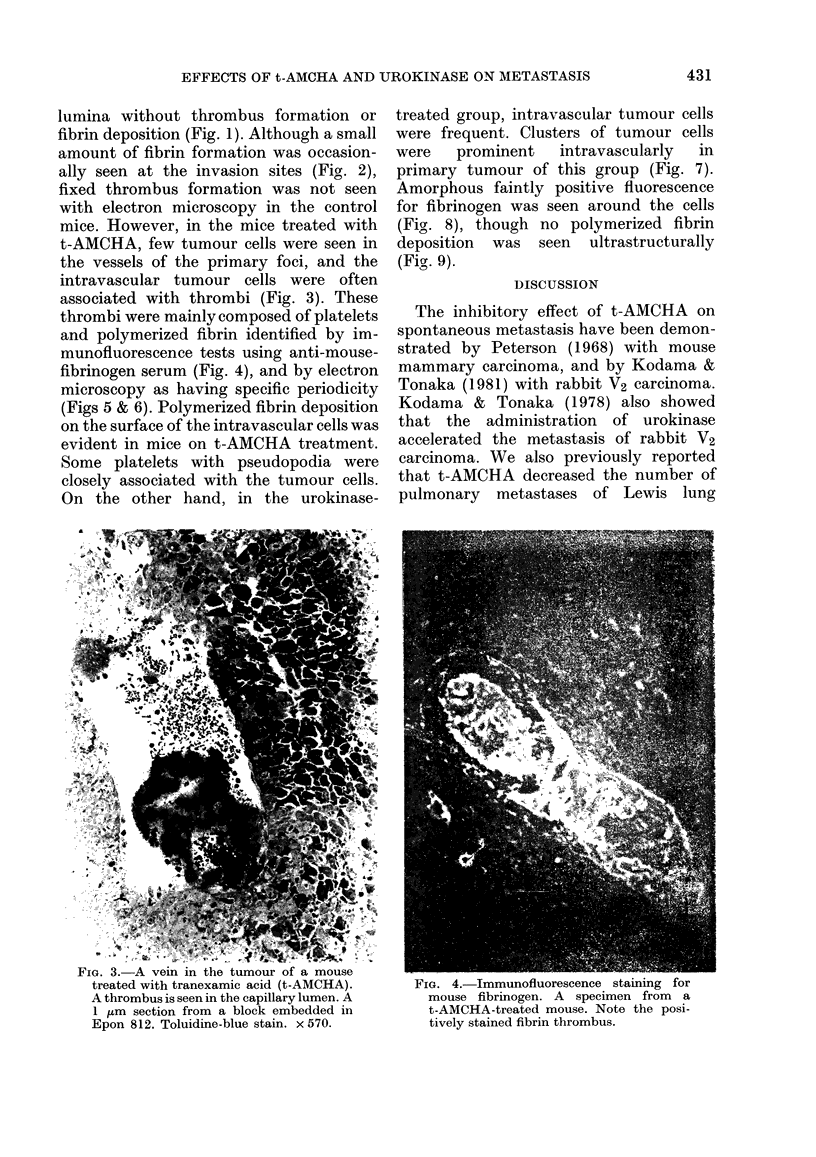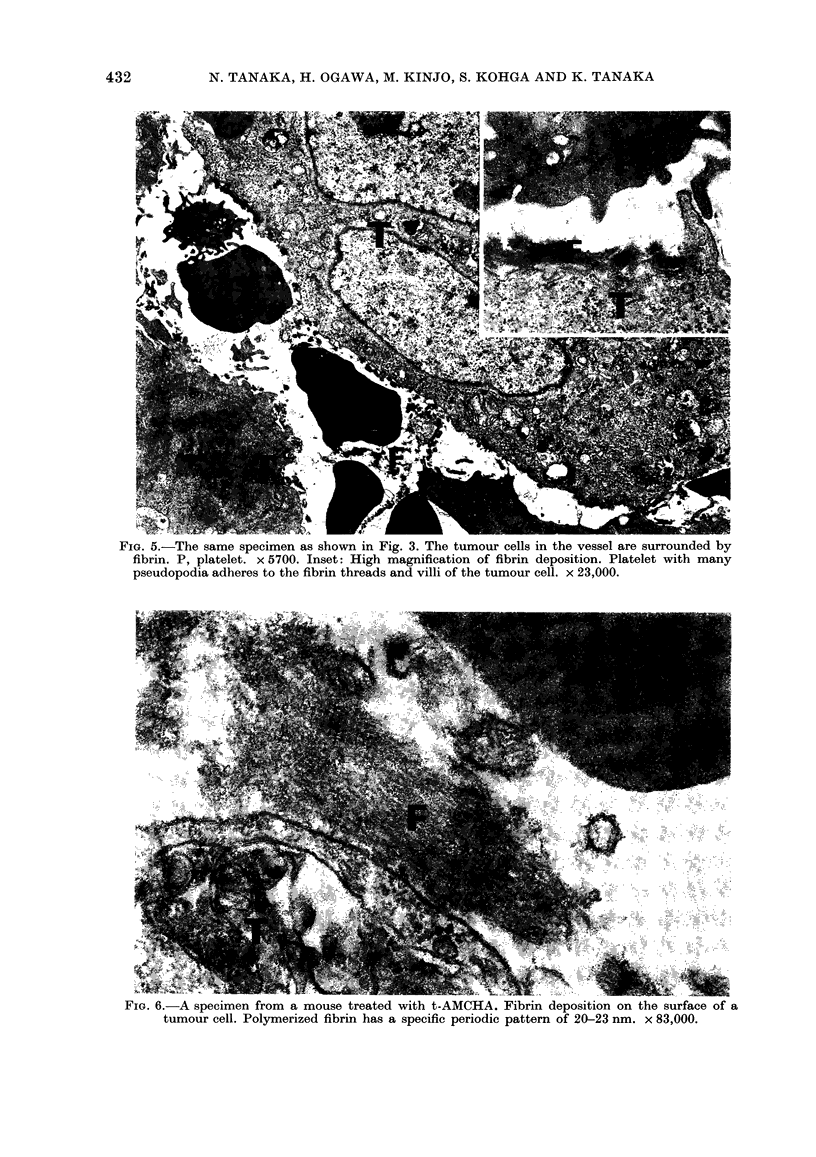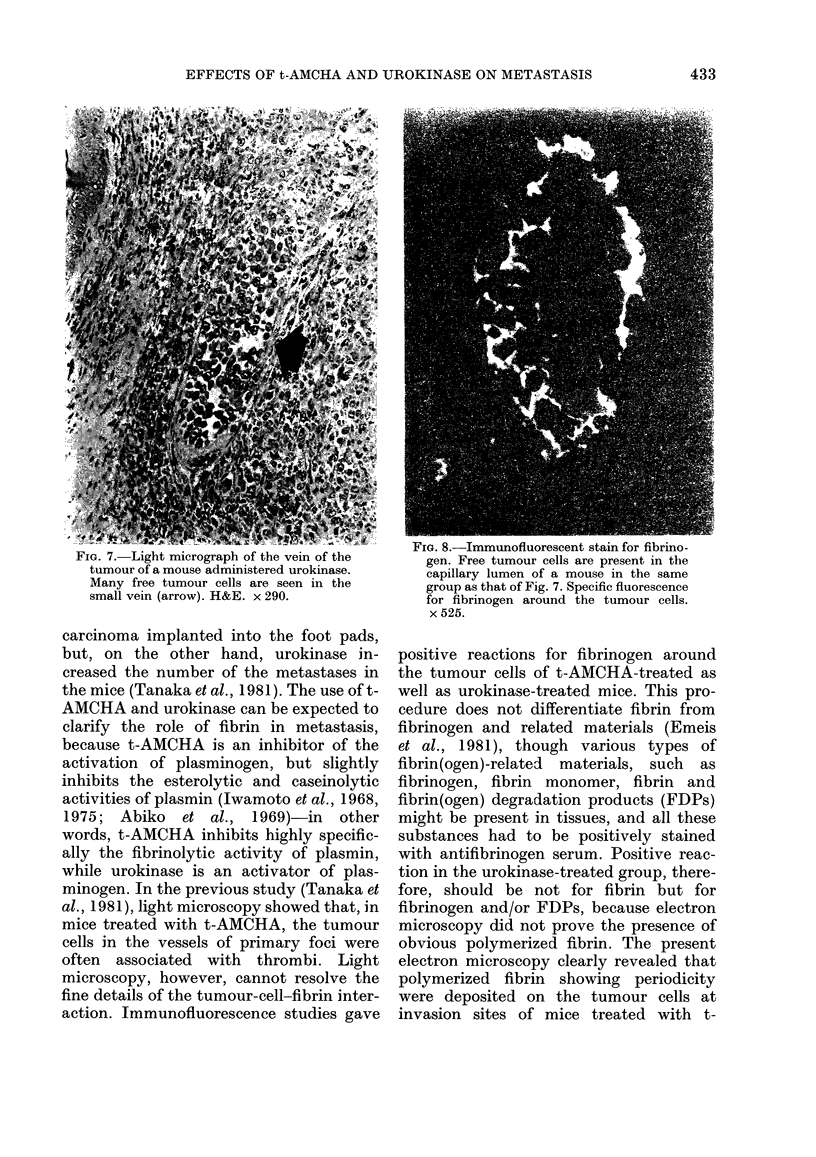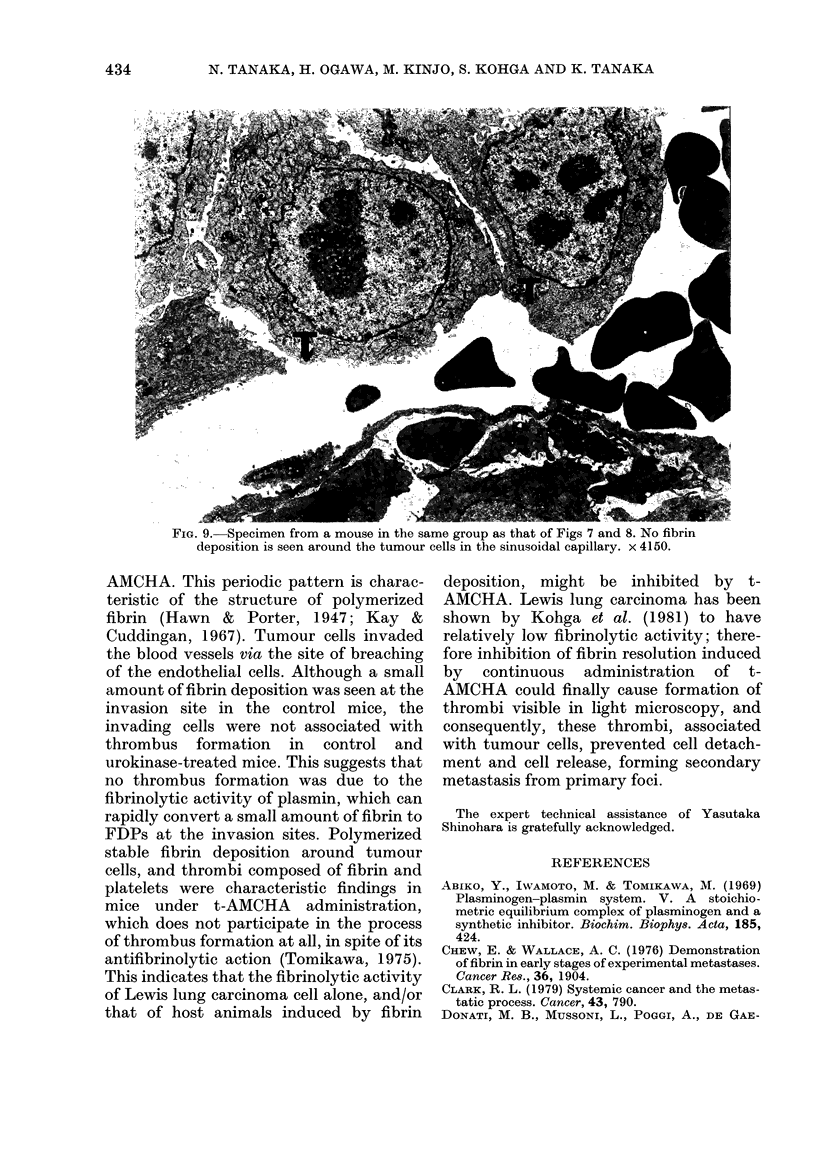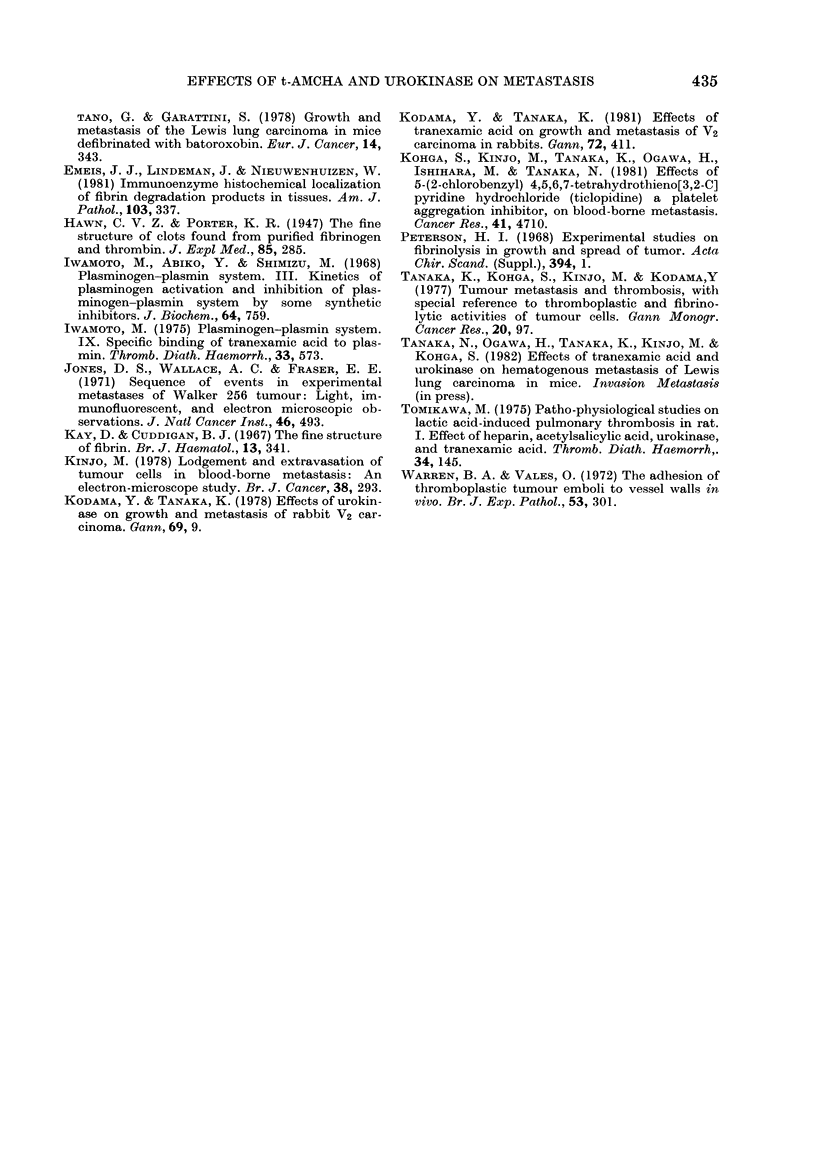# Ultrastructural study of the effects of tranexamic acid and urokinase on metastasis of Lewis lung carcinoma.

**DOI:** 10.1038/bjc.1982.220

**Published:** 1982-09

**Authors:** N. Tanaka, H. Ogawa, M. Kinjo, S. Kohga, K. Tanaka

## Abstract

**Images:**


*Br. J. Cancer* (1982) **46**, 428

### ULTRASTRUCTURAL STUDY OF THE EFFECTS OF TRANEXAMIC ACID AND UROKINASE ON METASTASIS OF LEWIS LUNG CARCINOMA

N. TANAKA*, H. OGAWA*, M. KINJO†, S. KOHGA‡ AND K. TANAKA†

*From the *Department of Pathology, Research Institute, Daiichi Seiyaku Co., Edogawa-ku, Tokyo* 134, *the †Department of Pathology, Faculty of Medicine, Kyushu University, Fukuoka, and the ‡Department of Pathology, Miyazaki Medical College, Miyazaki, Japan*

Received 26 January 1982 Accepted 21 April 1982

**Summary.—Lewis lung carcinoma cells were implanted in the foot-pads of mice and the effects of the plasminogen–plasmin inhibitor tranexamic acid (t-AMCHA) and of the plasminogen activator urokinase on metastasis were examined by electron microscopy. The intravascular tumour cells were not associated with thrombus formation in either control or urokinase-treated mice. Polymerized fibrin deposition around tumour cells and thrombi composed of fibrin and platelets was observed only in the mice given t-AMCHA. This suggests that the inhibition of fibrinolysis by tACC caused fibrin deposition and thrombus formation around intravascular tumour cells, which prevented release of the cells from primary foci to form secondary tumours. On the other hand, fibrinolysis induced by urokinase prevented thrombus formation, and accelerated cell release from primary foci.**

HAEMATOGENOUS METASTASIS is a continuous process consisting of several stages: (i) growth of primary tumours, (ii) invasion of tumour cells into the vessels, (iii) release and circulation of tumour cells and (iv) lodgement and secondary growth in distant organs. Several reports (Peterson, 1968; Tanaka *et al.*, 1977; Clark, 1979; Donati *et al.*, 1978) have suggested that the coagulation–fibrinolysis system plays different roles in the various stages of metastasis. Some workers (Jones *et al.*, 1971; Warren & Vales, 1972; Chew & Wallace, 1976; Kinjo, 1978) have demonstrated the association of fibrin with tumour-cell emboli in distant organs. Fibrin formation may aid in tumour arrest and adherence to blood vessel endothelium. On the other hand, tumour-cell release from primary foci is prior to cell lodgement in the destination organs. However, tumour-cell–fibrin interaction in this stage is still unclear for lack of adequate models. The previous study (Tanaka *et al.*, 1981) showed that the plasminogen–plasmin inhibitor trans-4-aminomethyl cyclohexane-1-carboxylic acid (t-AMCHA, tranexamic acid) decreased, and the plasminogen activator urokinase increased, the number of pulmonary metastases of Lewis lung carcinoma. These effects were considered to be mediated by the prevention and enhancement of cell release, due to t-AMCHA and urokinase, respectively.

The purpose of the present study is to clarify the effects of t-AMCHA and urokinase on the metastasis of Lewis lung carcinoma. Tumour-cell invasion sites were examined electron-microscopically, with special reference to tumour-cell–fibrin interaction.

### MATERIALS AND METHODS

Tranexamic acid (trans-4-aminomethyl cyclohexane-1-carboxylic acid) (Transamin, Daiichi Seiyaku Co. Ltd, Tokyo, Japan, t-AMCHA) and urokinase (Uronase, Mochida

Correspondence to Noriko Tanaka, Research Institute, Daiichi Seiyaku Co. Ltd, 16–13, Kitakasai-1-chome, Edogawaku, Toyko 134, Japan.

Pharmaceutical Co. Ltd, Tokyo, Japan, UK) were used.

The experimental procedure has previously been described (Tanaka *et al.*, 1981). Lewis lung carcinoma was passaged in male C57BL/6 mice (Charles River Japan Inc., Atsugi, Japan). The gently homogenized cells were filtered using 150 platinum mesh and suspended in Hanks' balanced salt solution at the rate of $10^6$/ml, and 0·1 ml of the suspension was inoculated into the foot pads of 10 female BDF1 mice (C57BL/6 $\times$ DBA/2) in each group; the viability of the tumour cells was 30% according to the trypan-blue test. The tumour-bearing legs were amputated 12 days after inoculation and the mice were necropsied 23 days after transplantation. Administrations of urokinase and t-AMCHA were begun 1 day before inoculation and continued until the day of amputation: twice-daily i.v. injections of urokinase at a dosage of 10,000 u/kg, and oral administration of t-AMCHA in the food, equivalent to a dose of 3 g/kg a day. Untreated mice inoculated with the same number of cells were used as the control.

On Day 12, the excised tumours were fixed in 10% buffered formalin, processed for paraffin section, and stained with haematoxylin and eosin, or Weigert fibrin stain. A part of each tumour was frozen immediately after the operation. Then, cryostat sections were used for immunofluorescence studies, using anti-mouse-fibrinogen rabbit serum and fluorescein-conjugated anti-rabbit IgG goat serum (Miles, Elkhart) for observation of fibrin(ogen). The controls were obtained by applying PBS or non-immunized rabbit serum to the specimens, instead of anti-mouse fibrinogen rabbit serum. Each antiserum was diluted with PBS in the ratio 1:79 before use. Tumours of 5 animals from the control group, and from each of the groups treated with t-AMCHA and 10,000 u/kg of urokinase were cut into 1mm$^3$ blocks, fixed in 2·5% glutaraldehyde, and subjected to post-fixation in 2% osmium tetroxide in 0·2M phosphate buffer (pH 7·3). After fixation, the specimens were dehydrated by a graded ethanol series, and embedded in Epon 812. Five blocks per mouse, that is, 25 blocks from each group, were cut into ultra-thin sections, which were double-stained with uranyl acetate and lead citrate, and observed by Hitachi H-500 transmission electron microscope.

On Day 23, animals were examined for the development of metastases. The numbers of metastatic foci on the pleural surfaces of both lungs were counted with the naked eye. The data were statistically analysed by Krusskall–Wallis test.

### RESULTS

The mean numbers of pulmonary metastatic foci of 10 mice were $21 \cdot 2 \pm 2 \cdot 5$, $44 \cdot 1 \pm 2 \cdot 3$, and $7 \cdot 0 \pm 1 \cdot 9$ (mean $\pm$ s.e.) in the control, urokinase- and t-AMCHA-treated groups of mice respectively. Urokinase increased the number of metastatic foci ($P < 0 \cdot 05$). On the other hand, in the mice given t-AMCHA, the number of metastatic foci was significantly lower than in the untreated ($P < 0 \cdot 05$). Invasion of tumour cells into the vascular lumina was observed in the primary foci excised from the mice on the 12th day. Free tumour cells were frequent in the sinusoidal vessels but rare in the veins. Out of 30 vessels of each of 5 animals, the incidences of these with free tumour cells were 29, 40 and 20% in control, urokinase- and t-AMCHA-treated mice respectively. The highest incidence in the urokinase-treated group coincided with the largest number of metastatic foci in the lungs of this group. Thrombus formation associated with intravasated tumour cells was rare in the control (13·7% of the vessels containing tumour cells) and absent in the urokinase-treated animals, but frequent in the t-AMCHA-treated mice (45% of such vessels) (Table). In the tumours of the control mice, capillary endothelial cells were torn apart at the sites of invasion and tumour cells invaded the capillary.

TABLE.—*Effect of tranexamic acid and urokinase on thrombus formation in intravasation site*

| Treatment | No. of vessels with tumour cells associated with thrombi / Total with intravasated cells (%) |
|---|---|
| Control | 4/29 (4) |
| Urokinase | 0/40 (0) |
| t-AMCHA | 9/20 (45) |

For each treatment there were 5 mice producing 5 blocks from which 100 vessels were observed.

FIG. 1.—A small blood vessel in the tumour of a control mouse. The capillary endothelial lining has been breached and tumour cells are invading the capillary lumen without thrombus formation. T, tumour cell. × 3500.

FIG. 2.—Tumour-cell invasion site of a control mouse. A small amount of fibrin is visible. The endothelial cell has fully degenerated, whereas the tumour cells are maintaining their structure comparatively well. T, tumour cell; F, fibrin; E, endothelial cell. × 3350.

lumina without thrombus formation or fibrin deposition (Fig. 1). Although a small amount of fibrin formation was occasionally seen at the invasion sites (Fig. 2), fixed thrombus formation was not seen with electron microscopy in the control mice. However, in the mice treated with t-AMCHA, few tumour cells were seen in the vessels of the primary foci, and the intravascular tumour cells were often associated with thrombi (Fig. 3). These thrombi were mainly composed of platelets and polymerized fibrin identified by immunofluorescence tests using anti-mouse-fibrinogen serum (Fig. 4), and by electron microscopy as having specific periodicity (Figs 5 & 6). Polymerized fibrin deposition on the surface of the intravascular cells was evident in mice on t-AMCHA treatment. Some platelets with pseudopodia were closely associated with the tumour cells. On the other hand, in the urokinase-treated group, intravascular tumour cells were frequent. Clusters of tumour cells were prominent intravascularly in primary tumour of this group (Fig. 7). Amorphous faintly positive fluorescence for fibrinogen was seen around the cells (Fig. 8), though no polymerized fibrin deposition was seen ultrastructurally (Fig. 9).

### DISCUSSION

The inhibitory effect of t-AMCHA on spontaneous metastasis have been demonstrated by Peterson (1968) with mouse mammary carcinoma, and by Kodama & Tonaka (1981) with rabbit $V_2$ carcinoma. Kodama & Tonaka (1978) also showed that the administration of urokinase accelerated the metastasis of rabbit $V_2$ carcinoma. We also previously reported that t-AMCHA decreased the number of pulmonary metastases of Lewis lung

FIG. 3.—A vein in the tumour of a mouse treated with tranexamic acid (t-AMCHA). A thrombus is seen in the capillary lumen. A 1 μm section from a block embedded in Epon 812. Toluidine-blue stain. ×570.

FIG. 4.—Immunofluorescence staining for mouse fibrinogen. A specimen from a t-AMCHA-treated mouse. Note the positively stained fibrin thrombus.

Fig. 5.—The same specimen as shown in Fig. 3. The tumour cells in the vessel are surrounded by fibrin. P, platelet. ×5700. Inset: High magnification of fibrin deposition. Platelet with many pseudopodia adheres to the fibrin threads and villi of the tumour cell. ×23,000.

Fig. 6.—A specimen from a mouse treated with t-AMCHA. Fibrin deposition on the surface of a tumour cell. Polymerized fibrin has a specific periodic pattern of 20–23 nm. ×83,000.

FIG. 7.—Light micrograph of the vein of the tumour of a mouse administered urokinase. Many free tumour cells are seen in the small vein (arrow). H&E. × 290.

FIG. 8.—Immunofluorescent stain for fibrinogen. Free tumour cells are present in the capillary lumen of a mouse in the same group as that of Fig. 7. Specific fluorescence for fibrinogen around the tumour cells. × 525.

carcinoma implanted into the foot pads, but, on the other hand, urokinase increased the number of the metastases in the mice (Tanaka *et al.*, 1981). The use of t-AMCHA and urokinase can be expected to clarify the role of fibrin in metastasis, because t-AMCHA is an inhibitor of the activation of plasminogen, but slightly inhibits the esterolytic and caseinolytic activities of plasmin (Iwamoto *et al.*, 1968, 1975; Abiko *et al.*, 1969)—in other words, t-AMCHA inhibits highly specifically the fibrinolytic activity of plasmin, while urokinase is an activator of plasminogen. In the previous study (Tanaka *et al.*, 1981), light microscopy showed that, in mice treated with t-AMCHA, the tumour cells in the vessels of primary foci were often associated with thrombi. Light microscopy, however, cannot resolve the fine details of the tumour-cell–fibrin interaction. Immunofluorescence studies gave positive reactions for fibrinogen around the tumour cells of t-AMCHA-treated as well as urokinase-treated mice. This procedure does not differentiate fibrin from fibrinogen and related materials (Emeis *et al.*, 1981), though various types of fibrin(ogen)-related materials, such as fibrinogen, fibrin monomer, fibrin and fibrin(ogen) degradation products (FDPs) might be present in tissues, and all these substances had to be positively stained with antifibrinogen serum. Positive reaction in the urokinase-treated group, therefore, should be not for fibrin but for fibrinogen and/or FDPs, because electron microscopy did not prove the presence of obvious polymerized fibrin. The present electron microscopy clearly revealed that polymerized fibrin showing periodicity were deposited on the tumour cells at invasion sites of mice treated with t-

FIG. 9.—Specimen from a mouse in the same group as that of Figs 7 and 8. No fibrin deposition is seen around the tumour cells in the sinusoidal capillary. ×4150.

AMCHA. This periodic pattern is characteristic of the structure of polymerized fibrin (Hawn & Porter, 1947; Kay & Cuddingan, 1967). Tumour cells invaded the blood vessels *via* the site of breaching of the endothelial cells. Although a small amount of fibrin deposition was seen at the invasion site in the control mice, the invading cells were not associated with thrombus formation in control and urokinase-treated mice. This suggests that no thrombus formation was due to the fibrinolytic activity of plasmin, which can rapidly convert a small amount of fibrin to FDPs at the invasion sites. Polymerized stable fibrin deposition around tumour cells, and thrombi composed of fibrin and platelets were characteristic findings in mice under t-AMCHA administration, which does not participate in the process of thrombus formation at all, in spite of its antifibrinolytic action (Tomikawa, 1975). This indicates that the fibrinolytic activity of Lewis lung carcinoma cell alone, and/or that of host animals induced by fibrin deposition, might be inhibited by t-AMCHA. Lewis lung carcinoma has been shown by Kohga *et al.* (1981) to have relatively low fibrinolytic activity; therefore inhibition of fibrin resolution induced by continuous administration of t-AMCHA could finally cause formation of thrombi visible in light microscopy, and consequently, these thrombi, associated with tumour cells, prevented cell detachment and cell release, forming secondary metastasis from primary foci.

The expert technical assistance of Yasutaka Shinohara is gratefully acknowledged.

### REFERENCES

ABIKO, Y., IWAMOTO, M. & TOMIKAWA, M. (1969) Plasminogen–plasmin system. V. A stoichiometric equilibrium complex of plasminogen and a synthetic inhibitor. *Biochim. Biophys. Acta*, **185**, 424.

CHEW, E. & WALLACE, A. C. (1976) Demonstration of fibrin in early stages of experimental metastases. *Cancer Res.*, **36**, 1904.

CLARK, R. L. (1979) Systemic cancer and the metastatic process. *Cancer*, **43**, 790.

DONATI, M. B., MUSSONI, L., POGGI, A., DE GAE-

TANO, G. & GARATTINI, S. (1978) Growth and metastasis of the Lewis lung carcinoma in mice defibrinated with batoroxobin. *Eur. J. Cancer*, **14**, 343.

EMEIS, J. J., LINDEMAN, J. & NIEUWENHUIZEN, W. (1981) Immunoenzyme histochemical localization of fibrin degradation products in tissues. *Am. J. Pathol.*, **103**, 337.

HAWN, C. V. Z. & PORTER, K. R. (1947) The fine structure of clots found from purified fibrinogen and thrombin. *J. Expl Med.*, **85**, 285.

IWAMOTO, M., ABIKO, Y. & SHIMIZU, M. (1968) Plasminogen–plasmin system. III. Kinetics of plasminogen activation and inhibition of plasminogen–plasmin system by some synthetic inhibitors. *J. Biochem.*, **64**, 759.

IWAMOTO, M. (1975) Plasminogen–plasmin system. IX. Specific binding of tranexamic acid to plasmin. *Thromb. Diath. Haemorrh.*, **33**, 573.

JONES, D. S., WALLACE, A. C. & FRASER, E. E. (1971) Sequence of events in experimental metastases of Walker 256 tumour: Light, immunofluorescent, and electron microscopic observations. *J. Natl Cancer Inst.*, **46**, 493.

KAY, D. & CUDDIGAN, B. J. (1967) The fine structure of fibrin. *Br. J. Haematol.*, **13**, 341.

KINJO, M. (1978) Lodgement and extravasation of tumour cells in blood-borne metastasis: An electron-microscope study. *Br. J. Cancer*, **38**, 293.

KODAMA, Y. & TANAKA, K. (1978) Effects of urokinase on growth and metastasis of rabbit $V_2$ carcinoma. *Gann*, **69**, 9.

KODAMA, Y. & TANAKA, K. (1981) Effects of tranexamic acid on growth and metastasis of $V_2$ carcinoma in rabbits. *Gann*, **72**, 411.

KOHGA, S., KINJO, M., TANAKA, K., OGAWA, H., ISHIHARA, M. & TANAKA, N. (1981) Effects of 5-(2-chlorobenzyl) 4,5,6,7-tetrahydrothieno[3,2-C] pyridine hydrochloride (ticlopidine) a platelet aggregation inhibitor, on blood-borne metastasis. *Cancer Res.*, **41**, 4710.

PETERSON, H. I. (1968) Experimental studies on fibrinolysis in growth and spread of tumor. *Acta Chir. Scand.* (Suppl.), **394**, 1.

TANAKA, K., KOHGA, S., KINJO, M. & KODAMA,Y (1977) Tumour metastasis and thrombosis, with special reference to thromboplastic and fibrinolytic activities of tumour cells. *Gann Monogr. Cancer Res.*, **20**, 97.

TANAKA, N., OGAWA, H., TANAKA, K., KINJO, M. & KOHGA, S. (1982) Effects of tranexamic acid and urokinase on hematogenous metastasis of Lewis lung carcinoma in mice. *Invasion Metastasis* (in press).

TOMIKAWA, M. (1975) Patho-physiological studies on lactic acid-induced pulmonary thrombosis in rat. I. Effect of heparin, acetylsalicylic acid, urokinase, and tranexamic acid. *Thromb. Diath. Haemorrh,.* **34**, 145.

WARREN, B. A. & VALES, O. (1972) The adhesion of thromboplastic tumour emboli to vessel walls *in vivo*. *Br. J. Exp. Pathol.*, **53**, 301.